# ​Parcel feature data derived from Google Street View images for urban land use classification in Brooklyn, New York Cityfor urban land use classification in Brooklyn, New York Cityretain-->

**DOI:** 10.1016/j.dib.2017.04.002

**Published:** 2017-04-08

**Authors:** Weixing Zhang, Weidong Li, Chuanrong Zhang, Dean M. Hanink, Xiaojiang Li, Wenjie Wang

**Affiliations:** a Department of Geography, University of Connecticut, Storrs, CT 06269-4148, USA; b Center for Environmental Science and Engineering, University of Connecticut, Storrs, CT 06269-4148, USA; c Connecticut State Data Center, University of Connecticut, Storrs, CT 06269-4148, USA

**Keywords:** Google Street View, Urban land use classification, Parcel feature, Detected text

## Abstract

Google Street View (GSV) was used for urban land use classification, together with airborne light detection and ranging (LiDAR) data and high resolution orthoimagery, by a parcel-based method. In this data article, we present the input raw GSV images, intermediate products of GSV images, and final urban land use classification data that are related to our research article "Parcel-based urban land use classification in megacity using airborne LiDAR, high resolution orthoimagery, and Google Street View" (Zhang et al., 2017) [1]. More detail about other used data and our findings can be found in Zhang et al. (2017) [1].

**Specifications Table**

**Table:** 

Subject area	Geography, Urban planning
More specific subject areas	Urban land use classification, Remote sensing
Type of data	Google Street View (GSV), parcel boundary GIS dataset, street GIS dataset, land use map, Python code, and MATLAB code
How data was acquired	GSV images were obtained via Google Maps APIs. Parcel boundary GIS dataset was downloaded from NYC Department of City Planning. Street GIS dataset was downloaded from the New York State GIS Program Office
Data format	IMG, JPG, SHP, CSV, PY (Python code), M (MATLAB code)
Experimental factors	Image processing
Experimental features	Feature detection, text recognition, image classification
Data source location	Center part of Brooklyn, New York, USA
Data accessibility	Data is accessible in this article

**Value of the data**

• The data provide GSV images for 25,121 parcels in Brooklyn, New York City.
• The urban land use classification result can be used as reference data of urban land use change monitoring of the study area.
• These parcel-based GSV images are useful for other micro scale urban studies in the study areas, such as urban landscape and neighborhood environment.

## 1 Data

Mixed residential & commercial buildings are difficult to classify using general remote sensing technologies because they have a lot of common characteristics (e.g. building-relevant characteristics, parcel-relevant characteristics, and vegetation characteristics) with single-family houses and multi-family residential buildings [1]. Therefore, we extracted text information from Google Street View (GSV) images and used it in urban land use classification to better distinguish mixed residential & commercial buildings from residential buildings, because the former ones have shop signs but the latter ones do not have. The urban land use classification was conducted using a parcel-based approach with the Random Forest classifier, based on airborne light detection and ranging (LiDAR) data, high resolution orthoimagery (HRO) images, and GSV images. Thirteen parcel features were chosen as input variables to the classifier for land use classification according to related previous researches [2], [3], [4] and empirical considerations. Four parcel features were derived from GSV images: *length of detected text from fov 30 GSV image*, *length of detected text from fov 45 GSV image*, *length of detected text from fov 60 GSV image*, and *index of English words from all detected text from GSV images* (Table 1).

## 2 Experimental design, materials and methods

### 2.1 Data acquisition

**Fig. 1:**
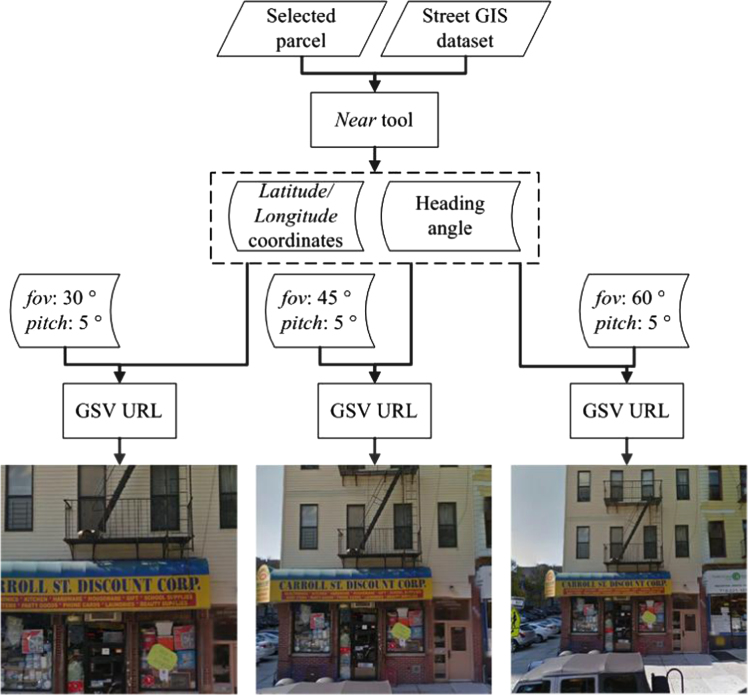
Google Street View (GSV) image acquisition. Using the *Near* tool to locate the nearest geo-location from Street Map and acquire the latitude and longitude coordinates and the heading angle for GSV image selection. Then GSV images with 3 different zoom degrees (i.e. 30°, 45°, and 60°) were downloaded by requesting GSV URL.

The parcel boundary GIS dataset was downloaded from the NYC Department of City Planning (DCP) with minor classes being merged with functionally similar major classes. The street GIS dataset downloaded from the New York State GIS Program Office was preprocessed to correct some out-of-date mistakes. The LiDAR dataset was downloaded in LAS format. ArcGIS 10.2 toolbox was applied to resample raw LiDAR data to 0.5 m in order to match the resolution of HRO images. The used HRO images were acquired from the USGS with a spatial resolution of 0.15 m and four channels (i.e. red, green, blue, and near infrared channels). A Python code was developed to automatically calculate the nearest geo-location from the street GIS dataset by reading the *x*- and *y*-coordinates of the geometric center of each parcel from the parcel boundary GIS dataset and capture the GSV images for that parcel by parsing GSV URL (Fig. 1).

### 2.2 Data processing

**Fig. 2:**
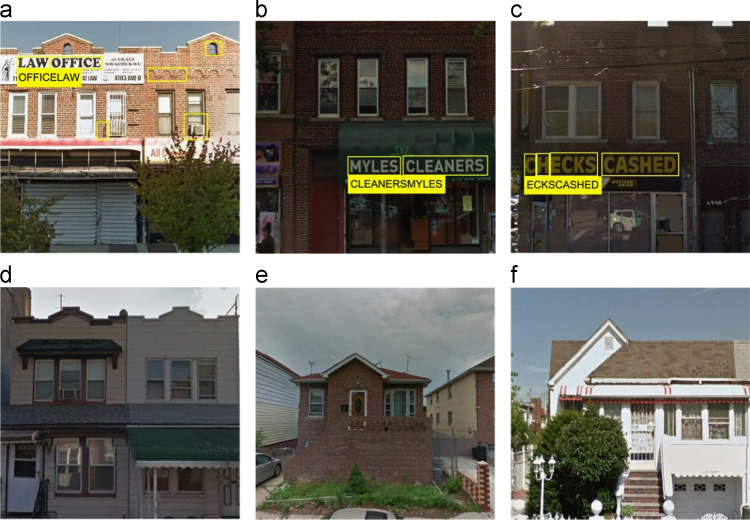
(a), (b), and (c): Google Street View (GSV) images for mixed residential & commercial buildings, which have detectable texts; (d), (e), and (f): GSV images for single-family houses and multi-family residential buildings, which do not have detectable texts.

Based on the assumptions that mixed residential & commercial buildings have shop signs and the shop signs can be detected and recognized as texts from the corresponding GSV images of parcels [1], mixed residential & commercial buildings (Fig. 2(a)–(c)) can be distinguished from single-family houses and multi-family residential buildings (Fig. 2(d)–(f)). The Computer Vision System Toolbox of MATLAB (Version R2016a) was employed to conduct fully automatic text detection and recognition from GSV images. Outputs of four parcel features from GSV images were written into a csv table as part of input variables, which include other nine common parcel features. To explore the use of GSV in separating parcels of mixed residential & commercial buildings from single-family houses and multi-family residential buildings, a comparison between the land use classification based on only the nine common parcel features (not including GSV-derived parcel features) and the land use classification based on all of the thirteen parcel features (including GSV-derived parcel features) were conducted by randomly selecting 20% of all parcels as the training sample data, with the random seeds 611, 1924, 3391, 6763, and 9930, respectively.

## Figures and Tables

**Table 1:** Description of selected parcel features derived from GSV images.

No.	Parcel feature	Description
1	Length of detected text from *fov* 30 GSV image	Length of detected text derived from requested GSV image with the horizontal field view angle being set to 30°
2	Length of detected text from *fov* 45 GSV image	Length of detected text derived from requested GSV image with the horizontal field view angle being set to 45°
3	Length of detected text from *fov* 60 GSV image	Length of detected text derived from requested GSV image with the horizontal field view angle being set to 60°
4	Index of English words from all detected text	Number of detected text being a English word from *fov* 30 GSV image, *fov* 30 GSV image, and *fov* 30 GSV image
